# The relationship between SARS-COV-2 RNA positive duration and the risk of recurrent positive

**DOI:** 10.1186/s40249-021-00831-6

**Published:** 2021-03-31

**Authors:** Hong Zhao, Chi Zhang, Xian-Xiang Chen, Qi Zhu, Wen-Xiang Huang, Yi-Lan Zeng, Ying-Xia Liu, Guo-Jun Li, Wei-Jun Du, Jing Yao, Jia-Wen Li, Peng Peng, Gui-Qiang Wang

**Affiliations:** 1 Department of Infectious Disease, Center for Liver Disease, Peking University First Hospital, Beijing, China; 2 Department of Infectious Disease, Peking University International Hospital, Beijing, China; 3 Administrative Office, Wuhan Pulmonary Hospital, Wuhan, China; 4 Department of Tuberculosis, Wuhan Pulmonary Hospital, Wuhan, China; 5 Department of Infectious Disease, The First Affiliated Hospital of Chongqing Medical University, Chongqing, China; 6 Administrative Office, Chengdu Public Health Clinical Center, Chengdu, China; 7 Administrative Office, The Third People’s Hospital of Shenzhen, Shenzhen, China; 8 Department of Hepatology III, The Third People’s Hospital of Shenzhen, Shenzhen, China; 9 Department of Internal Medicine, Fankou Branch of Ezhou Central Hospital, Ezhou, China; 10 Department of Obstetrics and Gynecology, Fankou Branch of Ezhou Central Hospital, Ezhou, China

**Keywords:** SARS-CoV-2, COVID-19, SARS-CoV-2 RNA positive duration, Recurrent positive, Prevention

## Abstract

**Background:**

The management of discharge COVID-19 patients with recurrent positive SARS-CoV-2 RNA is challenging. However, there are fewer scientific dissertations about the risk of recurrent positive. The aim of this study was to explore the relationship between SARS-COV-2 RNA positive duration (SPD) and the risk of recurrent positive.

**Methods:**

This case–control multi-center study enrolled participants from 8 Chinese hospital including 411 participants (recurrent positive 241). Using unadjusted and multivariate-adjusted logistic regression analyses, generalized additive model with a smooth curve fitting, we evaluated the associations between SPD and risk of recurrent positive. Besides, subgroup analyses were performed to explore the potential interactions.

**Results:**

Among recurrent positive patients, there were 121 females (50.2%), median age was 50 years old [interquartile range (IQR): 38–63]. In non-adjusted model and adjusted model, SPD was associated with an increased risk of recurrent positive (fully-adjusted model: *OR* = 1.05, 95% *CI*: 1.02–1.08, *P* = 0.001); the curve fitting was not significant (*P* = 0.286). Comparing with SPD < 14 days, the risk of recurrent positive in SPD > 28 days was risen substantially (*OR* = 3.09, 95% *CI*: 1.44–6.63, *P* = 0.004). Interaction and stratified analyses showed greater effect estimates of SPD and risk of recurrent positive in the hypertension, low monocyte count and percentage patients (*P* for interaction = 0.008, 0.002, 0.036, respectively).

**Conclusion:**

SPD was associated with a higher risk of recurrent positive and especially SPD > 28 day had a two-fold increase in the relative risk of re-positive as compared with SPD < 14 day. What’s more, the risk may be higher among those with hypertension and lower monocyte count or percentage.

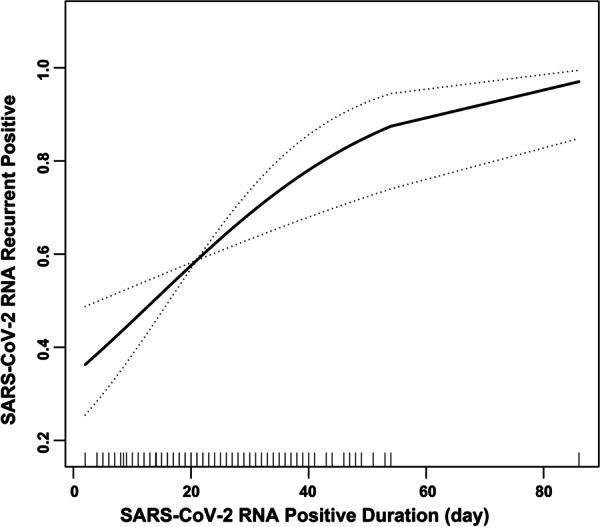

**Supplementary Information:**

The online version contains supplementary material available at 10.1186/s40249-021-00831-6.

## Background

At the end of 2019, an unexplained pneumonia occurred which was quickly identified and named coronavirus disease 2019 (COVID-19) [caused by severe acute respiratory syndrome coronavirus 2 (SARS-CoV-2)] [1]. As of December 15, 2020, SARS-CoV-2 has infected more than 73 million and the death exceeded 1.6 million worldwide [2]. Currently, there are approximately 500 000 new confirmed patients daily, posing huge challenges for public health and medical institutions [2].

At present, COVID-19 recovered patients have more than 50 million worldwide [2], and most of the infected people have lost the virus within 3 weeks [3–5]. However, there are numerous reports that some patients are recurrent positive [6–10]. Unfortunately, the mechanism leading to these re-positive cases is still unclear. The reasons may be complex and varied, including false-negative, false-positive RT-PCR tests; reactivation; and re-infection with SARS-CoV-2 [11, 12]. Yang’s study involving 93 re-positive patients showed that 72% (67/93) of the re-positive patients were clinically classified as asymptomatic infection, and the median of viral RNA level in recurrent-positive patients was 3.2 log10 copies/ml (ranged from 1.8 to 5.7) [5]. Another study involving 420 patients showed that 45.2% (190/420) of re-positive patients were asymptomatic [8]. As the condition of asymptomatic infection was hidden, the identification of risk factors of recurrent positive was one of the key points of COVID-19 prevention and control [13].

To our knowledge, most current research on COVID-19 focuses on the epidemiology, clinical features and treatment, but not on viral RNA shedding and risk of recurrent positive. Here, we discuss the relationship between them in a large patient cohort.

## Methods

### Study design and participants

A case–control multi-center study was performed in patients with COVID-19 hospitalized from January to June 2020 at six hospitals (Wuhan, Chongqing Shenzhen, Ezhou) in China. A control cohort with COVID-19 but without recurrent positive was identified from the aforementioned hospitals and preliminarily matched by age ± 5 years, sex.

Both cases and controls were restricted to the discharge of COVID-19 criteria [14]: (1) No fever for more than three days. (2) Respiratory symptoms significantly improved. (3) Pulmonary imaging showed that acute exudative lesions were significantly absorbed and improved. (4) The SARS-CoV-2 RNA test of respiratory tract samples was negative for two consecutive times (with samples taken at least 24 h apart).

The trial was done in accordance with the principles of the Declaration of Helsinki and the International Conference on Harmonization–Good Clinical Practice guidelines. This study has been approved by the Ethics Committee of Peking University First Hospital (2020-056) and waived informed consent because data were deidentified.

### Definition of variables

All cases were confirmed by laboratory and RT-PCR confirmed the presence of SARS-CoV-2 RNA in pharyngeal swabs. The end of virus RNA shedding was judged by more than two continuous negative RT-PCR results. Clinical classification of COVID-19 according to Chinese COVID-19 prevention and treatment guidelines (8th edition) (Additional file 1: Table S1) [14]. The definition of chronic diseases (see Table 1) involved in this study is as follows: chronic pulmonary disease (CPD) included chronic obstructive pulmonary disease, tuberculosis, asthma, and idiopathic pulmonary fibrosis; chronic liver disease (CLD) included chronic hepatitis B, chronic hepatitis C, nonalcoholic steatohepatitis, autoimmune liver disease and cirrhosis. Antiviral drugs (see Table 1) include Favipiravir (Haizheng Pharmaceutical Co. Ltd, Taizhou, China), Oseltamivir (Dongyangguang Pharmaceutical Co. Ltd, Yichang, China), Remdesivir (Gilead Sciences, California, USA) Chloroquine/Hydroxychloroquine (SHANGHAI PHARMA, Shanghai, China), Lopinavir–Ritonavir (AbbVie Inc. North Chicago, Illinois, U.S.A.), and Arbidol (CSPC PHARMA, Shijiazhuang, China).

**Table 1:** Baseline Characteristics of All Participants

	Non-recurrent positive	Recurrent positive	*P* value
No. of patients	170	241	
Sex			
Female	81 (47.65%)	121 (50.21%)	0.609
Male	89 (52.35%)	120 (49.79%)	
Age, Median (IQR), year	47.00 (35.00–61.00)	50.00 (38.00–63.00)	0.023
BMI, Mean (SD), kg/m^2^	22.93 (3.24)	22.98 (4.35)	0.894
SPD, Median (IQR), day	16.50 (12.00–24.00)	20.00 (13.00–29.00)	0.020
Routine blood test			
WBC, Mean (SD), × 10^9^/L	5.37 (1.89)	6.22 (2.69)	< 0.001
NC, Median (IQR), × 10^9^/L	2.86 (2.15–3.91)	3.10 (2.36–4.18)	0.163
NP, Mean (SD), %	58.69 (12.60)	54.90 (17.74)	0.017
LC, Median (IQR), × 10^9^/L	1.34 (1.00–1.80)	1.63 (1.24–2.12)	< 0.001
LP, Mean (SD), %	29.73 (11.86)	31.71 (14.66)	0.146
MC, Median (IQR), × 10^9^/L	0.49 (0.39–0.63)	0.43 (0.34–0.56)	0.005
MP, Median (IQR), %	9.75 (7.53–12.50)	7.40 (6.10–9.40)	< 0.001
Hemoglobin, Mean (SD), g/L	130.98 (16.20)	128.03 (21.38)	0.130
PLT, Mean (SD), × 10^9^/L	203.06 (66.38)	219.19 (74.74)	0.025
Clinical type			
Mild	7 (4.12%)	22 (9.13%)	0.141
Moderate	141 (82.94%)	179 (74.27%)	
Severe	17 (10.00%)	32 (13.28%)	
Critical	5 (2.94%)	8 (3.32%)	
Underlying disease			
No. of chronic diseases			
0	123 (72.35%)	139 (57.68%)	0.002
1	35 (20.59%)	65 (26.97%)	
2	12 (7.06%)	21 (8.71%)	
3	0 (0.00%)	12 (4.98%)	
4	0 (0.00%)	4 (1.66%)	
Hypertension			
No	144 (84.71%)	191 (79.25%)	0.161
Yes	26 (15.29%)	50 (20.75%)	
Diabetes			
No	157 (92.35%)	218 (90.46%)	0.503
Yes	13 (7.65%)	23 (9.54%)	
CHD			
No	166 (97.65%)	227 (94.19%)	0.092
Yes	4 (2.35%)	14 (5.81%)	
CPD			
No	165 (97.06%)	225 (93.36%)	0.094
Yes	5 (2.94%)	16 (6.64%)	
CKD			
No	168 (98.82%)	238 (98.76%)	0.95
Yes	2 (1.18%)	3 (1.24%)	
CLD			
No	162 (95.29%)	203 (84.23%)	< 0.001
Yes	8 (4.71%)	38 (15.77%)	
Malignant tumor			
No	168 (98.82%)	236 (97.93%)	0.705
Yes	2 (1.18%)	5 (2.07%)	
Treatment			
No. of antiviral drugs			
0	32 (18.82%)	46 (19.09%)	0.001
1	72 (42.35%)	106 (43.98%)	
2	47 (27.65%)	84 (34.85%)	
3	19 (11.18%)	5 (2.07%)	
Glucocorticoid			
No	143 (84.12%)	223 (92.53%)	0.007
Yes	27 (15.88%)	18 (7.47%)	

Data presented as mean and standard deviation (Gaussian distribution, compared with student *t*-test) or median and quartile (Skewed distribution, compared with Kruskal–Wallis analysis) for continuous variables; number and percentage for categorical variables (Chi-square or Fisher’s exact tests)

Clinical type, routine blood test and treatment were the conditions of previous discharge

CPD included COPD, tuberculosis, asthma, and idiopathic pulmonary fibrosis. CLD included chronic hepatitis B, chronic hepatitis C, nonalcoholic steatohepatitis, autoimmune liver disease and cirrhosis. Antiviral drugs include Favipiravir, Oseltamivir, Chloroquine/Hydroxychloroquine, Lopinavir–Ritonavir, Remdesivir and Arbidol

*SD* standard deviation, *CHD* coronary heart disease, *CPD* chronic pulmonary disease, *CKD* chronic kidney disease, *CLD* chronic liver disease, *WBC* white blood cell count, *NC* neutrophil count, *NP* neutrophil percentage, *LC* lymphocyte count, *LP* lymphocyte percentage, *MC* monocyte count, *MP* monocyte percentage, *PLT* platelet count, *SPD* SARS-CoV-2 RNA positive duration

### Statistical analysis

Data are reported as mean (standard deviation, SD) (Gaussian distribution) or median (interquartile range; Q1–Q3) (Skewed distribution) for continuous variables and as numbers (percentages) for categorical variables. Chi-square or Fisher’s exact tests (categorical variables); student *t*-test (normal distribution) or Man-Whitney U test (skewed distribution) were used to detect the differences among recurrent positive (binary variable).

Our statistical analyses consisted of three main steps. In Step 1, according to the recommendation of STROBE statement [15], to examine the correlation between SARS-CoV-2 RNA positive duration (SPD) and risk of recurrent positive, we constructed three distinct models using univariate and multivariate binary logistic regression model, including non-adjusted model (no covariates were adjusted), minimally-adjusted model (only sex and age were adjusted) and fully-adjusted model (covariates presented in Table 1 were adjusted). Effect sizes with 95% confidence intervals were recorded. In Step 2, we also use the generalized additive model (GAM) and the smooth curve fitting (penalized spline method) to explore whether there is a non-linear relationship between SPD and recurrent positive. Besides, two-piecewise binary logistic regression model was also used to explain the nonlinearity further. In Step 3, the subgroup analyses were performed using stratified binary logistic regression model. For continuous variable, we first converted it to a categorical variable according to the clinical cut point or tertile, and then performed an interaction test. Tests for effect modification for those of subgroup indicators were followed by the likelihood ration test. To avoid the adverse effect resulting from selection bias and unavailable information, we used multiple imputation by chained equations to impute missing covariate date. In imputed data, we performed a sensitivity analyses to test whether imputed data can change the distribution of covariates [16].

To text the robustness of our results, we performed a sensitivity analysis. We converted SPD into a categorical variable according to the bisected, and calculated the P for trend in order to verify the results of SARS-CoV-2 RNA positive duration as the continuous variable, and to examine the possibility of nonlinearity. 

Modeling was performed with the statistical software packages R (http://www.R-project.org, The R Foundation) and EmpowerStats (http://www.empowerstats.com, X&Y Solutions, Inc, Boston, MA). *P* values less than 0.05 (two-sided) were considered statistically significant.

## Results

### Baseline characteristics of patients

From January to June, 2020, we collected the demographic and clinical data of 241 recurrent positive patients after discharged from the above-mentioned 6 hospitals. For missing of covariates, the data distribution does not change before and after imputations (Additional file 1: Table S2, S3). Baseline characteristics were listed in Table 1. Among them, there were 121 females (50.2%), the median age was 50 years old (IQR: 38–63), and 79 (32.8%) had BMI over 24 kg/m^2^. Clinical classification, blood routine examination and treatment were all the results of the previous hospitalization. The clinical type was mainly moderate, accounting for 74.3% (179/241). There were 102 (42.3%) with at least one underlying disease, of which hypertension (20.8%, 50/241) was the most common, followed by chronic liver disease (15.8%, 38/241). In terms of treatment, about 80% (195/241) of patients have used at least one antiviral drug, and 7.5% (18/241) have used corticosteroids.

There were no significant differences between recurrent positive and non-recurrent positive patients in terms of sex (*P* = 0.609), BMI (*P* = 0.894), clinical severity (*P* = 0.141) on first admission. In terms of blood routine test, there was no statistical difference in neutrophil count (*P* = 0.163), lymphocyte percentage (*P* = 0.146) and hemoglobin (*P* = 0.130) before discharge. However, the white blood cell count (*P* < 0.001), lymphocyte count (*P* < 0.001) and platelet count (*P* = 0.025) in the recurrent positive group were significantly higher than non-recurrent positive group, while the neutrophil percentage (*P* = 0.017), monocyte count (*P* = 0.005) and monocyte percentage (*P* < 0.001) were exact converse (Table 1). More than 40% of patients in the recurrent positive group had at least one underlying disease, compared with less than 30% in the non-recurrent positive group (42.32% vs 28.65%, *P* = 0.002). There are also some differences in the previous hospitalization treatment between the two groups, as detailed in Table 1.

### The relationship between SPD and risk of recurrent positive

We used univariate linear regression model to evaluate the associations between SPD and the risk of recurrent positive. Meanwhile, we showed the non-adjusted and adjusted models in Table 2. In non-adjusted model, SPD was positively associated with an increased risk for recurrent positive (*OR* = 1.03, 95% *CI*: 1.01–1.04, *P* = 0.009). In minimally-adjusted model (adjusted age, sex), the result did not have obvious changes (*OR* = 1.02, 95% *CI*: 1.01–1.04, *P* = 0.013). In fully-adjusted model (adjusted age, sex and other covariates presented in Table 1), the association between SPD and recurrent positive risk had a similar trend, but with a slightly raised magnitude (*OR* = 1.05, 95% *CI*: 1.02–1.08, *P* = 0.001). In the fully-adjusted model, compared with SPD less than 14 days, there was no significant difference in SPD 14–28 days (*OR* = 1.25, 95% *CI*: 0.68–2.30, *P* = 0.476), while the risk of recurrent positive in SPD more than 28 days was risen substantially (*OR* = 3.09, 95% *CI*: 1.44–6.63, *P* = 0.004). For the purpose of sensitivity analysis, we also handled SPD as a categorical variable (tertile) and found the same trend (p for the trend was 0.005).

**Table 2:** Relationship between previous SARS-CoV-2 RNA positive duration and recurrent positive in different models

Variable	Non-adjusted model			Minimally-adjusted model			Fully-adjusted model		
	*OR*	95% *CI*	*P*	*OR*	95% *CI*	*P*	*OR*	95% *CI*	*P*
SPD (day)	1.03	1.01, 1.04	0.009	1.02	1.01–1.04	0.013	1.05	1.02–1.08	0.001
SPD (day) (tertile)									
< 14	Ref	-	-	Ref	-	-	Ref	-	-
14–28	1.15	0.72–1.83	0.564	1.11	0.69–1.78	0.663	1.25	0.68–2.30	0.476
≥ 28	1.68	0.97–2.92	0.067	1.6	0.91–2.80	0.102	3.09	1.44–6.63	0.004
P for trend	-	-	0.071	-	-	0.108	-	-	0.005

Non-adjusted model: we did not adjust other covariates

Minimally-adjusted model: we adjusted age and sex

Fully adjusted model: we adjusted age, sex and other covariates presented in Table 1

*SPD* SARS-CoV-2 RNA positive duration, *CI* confidence interval, *OR* odd ratio, *Ref.* reference

### The analysis of non-linear relationship between SPD and risk of recurrent positive

**Fig. 1:**
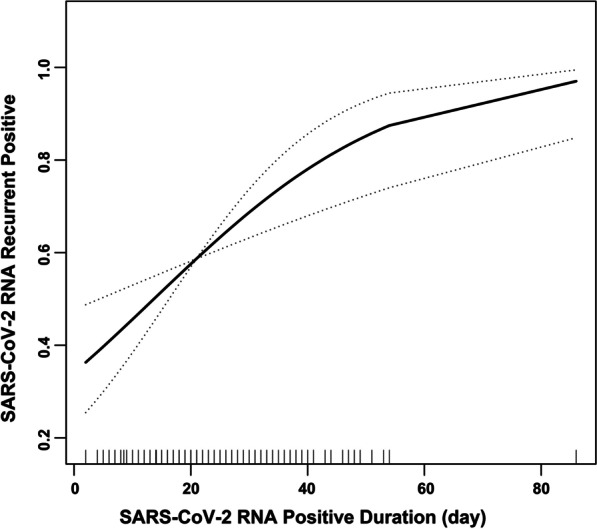
Multivariate adjusted smoothing spline plots of SARS-CoV-2 RNA positive in previous hospitalization and recurrent positive. We adjusted age, sex and other covariates presented in Table 1. The solid line represents the best-fit line, and the dotted lines are 95% confidence intervals. SARS-CoV-2: Severe acute respiratory syndrome coronavirus 2

Because SPD is a continuous variable, we still need curve fitting to explore whether there is a non-linear relationship between SPD and risk of recurrent positive (although the previous linear regression results are relatively robust). Under the fully-adjusted model, there seems to be a non-linear relationship between SPD and risk of recurrent positive from my subjective point of view (Fig. 1). By using a two-piecewise linear regression model, we calculated that the inflection point was 8. On the left of the inflection point, the *OR* (95% *CI*) and *P* value were 1.35 (0.83–2.19) and 0.228, respectively. On the right of the inflection point, the *OR* (95% *CI*) and *P* value were 1.04 (1.02–1.07) and 0.002, respectively. However, compared with the linear model, the difference is not statistically significant (*P* for log likelihood ratio test was 0.286) (Table 3).

**Table 3:** The result of two-piecewise linear regression model

	*OR* (95% *CI*)	*P* value
Fitting model by standard linear regression	1.05 (1.02–1.08)	0.001
Fitting model by two-piecewise linear regression		
Inflection point of virus positive duration (day)	8	-
< 8	1.35 (0.83–2.19)	0.228
≥ 8	1.04 (1.02–1.07)	0.002
*P* for log likelihood ratio test	-	0.286

We adjusted age, sex and other covariates presented in Table 1

*CI* confidence interval, *OR* odd ratio

### Subgroup analysis of SARS-CoV-2 RNA recurrent positive

As is shown in Table 4, the test for interactions were significant for monocyte count and percentage (*P* for interaction = 0.002, 0.036, respectively). Both monocyte count and percentage showed that the lower the value, the higher the risk of recurrent positive. What’s more, hypertension was also significant (*P* for interaction = 0.008). Hypertension increases the risk of recurrent positive. From the statistical point of view, lymphocyte count and the use of glucocorticoids also showed interaction. While the test for interactions were not statistically significant for demographic and other clinical data (*P* for interaction > 0.05).

**Table 4:** Subgroup analysis of SARS-CoV-2 RNA recurrent positive

Subgroup	No. of participants	*OR*	95% *CI*	*P* value	*P* for interaction
Sex					
Female	202	1.05	1.01–1.09	0.011	0.789
Male	209	1.06	1.01–1.10	0.018	
Age (year)					
< 18	14	-	-	-	-
≥ 18	397	1.05	1.02–1.08	0.001	
BMI (kg/m^2^)					
< 24	267	1.04	1.00–1.07	0.026	0.350
≥ 24	144	1.07	1.02–1.12	0.007	
Clinical type					
Mild-to-moderate infection	349	1.05	1.02–1.09	< 0.001	0.080
Severe-to-critical infection	62	0.94	0.84–1.05	0.289	
White blood cell count (tertile)					
Low (2.70–4.76)	134	1.09	1.04–1.15	0.001	0.176
Middle (4.80–6.30)	140	1.03	0.99–1.07	0.144	
High (6.31–36.10)	137	1.05	0.99–1.10	0.115	
Neutrophil count (tertile)					
Low (0.47–2.53)	133	1.10	1.03–1.17	0.004	0.223
Middle (2.55–3.65)	141	1.03	0.99–1.08	0.176	
High (3.66–13.45)	137	1.07	1.01–1.13	0.023	
Neutrophil percentage (tertile)					
Low (7.2–53.7)	137	1.00	0.95–1.06	0.899	0.112
Middle (53.8–64.0)	137	1.09	1.02–1.17	0.011	
High (64.1–94.9)	137	1.06	1.00–1.13	0.052	
Lymphocyte count (tertile)					
Low (0.16–1.27)	137	1.04	0.98–1.10	0.172	0.023
Middle (1.28–1.78)	132	1.14	1.06–1.23	0.001	
High (1.80–8.22)	142	1.02	0.97–1.07	0.382	
Lymphocyte percentage (tertile)					
Low (4.4–24.9)	137	1.04	0.98–1.11	0.153	0.738
Middle (25.0–33.6)	137	1.06	1.01–1.12	0.015	
High (33.7–84.0)	137	1.03	0.98–1.09	0.193	
Monocyte count (tertile)					
Low (0.03–0.38)	135	1.20	1.08–1.32	0.001	0.002
Middle (0.39–0.53)	133	1.03	0.96–1.11	0.343	
High (0.54–2.66)	143	1.00	0.94–1.06	0.989	
Monocyte percentage (tertile)					
Low (0.3–7.1)	134	1.15	1.05–1.26	0.003	0.036
Middle (7.2–9.7)	138	1.10	1.01–1.21	0.028	
High (9.8–41.7)	139	1.01	0.94–1.08	0.847	
Hemoglobin (tertile)					
Low (71–122)	136	1.09	1.01–1.17	0.018	0.673
Middle (122–137)	133	1.05	0.99–1.11	0.123	
High (137–297)	142	1.06	1.00–1.13	0.035	
Platelet count (tertile)					
Low (66–180)	137	1.04	0.98–1.11	0.191	0.871
Middle (180–226)	137	1.05	1.00–1.11	0.032	
High (226–577)	137	1.06	1.01–1.12	0.021	
Chronic diseases					
No	262	1.04	1.00–1.07	0.031	0.181
Yes	149	1.08	1.02–1.13	0.004	
Hypertension					
No	335	1.04	1.01–1.07	0.019	0.008
Yes	76	1.17	1.06–1.29	0.002	
Diabetes					
No	375	1.05	1.02–1.08	0.001	-
Yes	36	-	-	-	
Chronic pulmonary disease					
No	390	1.05	1.02–1.08	0.001	-
Yes	21	-	-	-	
Coronary heart disease					
No	393	1.05	1.02–1.07	0.001	-
Yes	18	-	-	-	
Chronic kidney disease					
No	406	1.05	1.02–1.08	0.001	-
Yes	5	-	-	-	
Chronic liver disease					
No	365	1.05	1.02–1.08	0.001	-
Yes	46	-	-	-	
Malignant tumor					
No	404	1.05	1.02–1.08	< 0.001	-
Yes	7	-	-	-	
No. of antiviral drugs					
0	78	1.10	0.97–1.25	0.133	0.688
1	178	1.05	1.00–1.10	0.030	
≥ 2	155	1.04	1.00–1.08	0.037	
Glucocorticoid					
No	366	1.04	1.01–1.06	0.005	0.005
Yes	45	1.78	0.80–3.94	0.158	

Clinical type, routine blood test and treatment were the conditions of the previous discharge

Because of the small number of cases in the Age and underlying disease (Diabetes, chronic pulmonary disease, coronary heart disease, chronic kidney disease, chronic liver disease, malignant tumor) subgroups, it is failed to calculate the effect value, confidence interval and interaction P value

Because 1 patient is chronic lymphoblastic leukemia, there are abnormal values at the maximum of white blood cell count, lymphocyte count and lymphocyte percentage

*CI* confidence interval, *OR* odd ratio

## Discussion

In this case–control study, we used GLM and GAM models to elucidate the relationship between SPD and risk of recurrent positive among participants, so as to predict and early-warn the high-risk of recurrent-positive. This is of great significance not only to the patient’s recovery after discharge, but also to reduce the risk of COVID-19 transmission. Whether in the non-adjusted model (*OR* = 1.03, 95% *CI*: 1.01–1.04), the minimally-adjusted model (*OR* = 1.02, 95% *CI*: 1.01–1.04) and the fully-adjusted model (*OR* = 1.05, 95% *CI*: 1.02–1.08), we discovered that the prolongation of SPD was associated with the increased risk of recurrent positive. When we handled SPD as a categorical variable, the same trend was also observed. Subsequently, we also explored whether there is a curvilinear relationship between SPD and recurrent positive, and the result is negative (*P* = 0.286). This once again proved the robustness of our results.

We conducted a PubMed search using the following search strategy: (“recurrent positive” [Title/Abstract] OR “re-positive” [Title/Abstract]) AND (“COVID-19” [Title/Abstract] OR “SARS-CoV-2” [Title/Abstract]). Although there is no direct study of the relationship between SPD and recurrent positive, several related studies have been found. A study of 30 recurrent positive patients from China showed that there was a significant difference in length of hospitalization between the recurrent positive group and the non- recurrent positive group [median and IQR 36 day (30–44) vs. 25 (19–34)] [17]. Although the study did not directly point out the difference in SPD between the two groups, there was a direct correlation between length of stay and SPD (Chinese discharge criteria are described in “Study design and participants” section, the most important of which was the test of SARS-CoV-2 RNA negative) [14]. In another study of 23 re-positive patients, the median (IQR) SPD was essentially consistent with the results of our study (19 day [14, 26], 20 [13–29], respectively) [18]. Unfortunately, they did not compare the difference between recurrent positive and non-recurrent positive group. However, one study results may inconsistent with our findings. Lu et al. reported that there was no association between the use of biomass fuels and hypertension based on 87 recurrent positive patients [19]. The onset-discharge time (median 17 day vs. 33, *P* < 0.001) and initial hospital stay (median 14 day vs. 28, *P* < 0.001) in the recurrent positive group were longer than those in the non- recurrent positive group. This may be related to the higher proportion of severe patients (23.8% vs. 0.0%) in the non-recurrent positive group in this study.

Subgroup analysis and interaction analysis are extremely important for a scientific study. In our sensitivity analysis, the risk of recurrent positive in patients with hypertension was significantly higher than without hypertension (*P* = 0.008). At present, there is a great controversy about the relationship between hypertension, use of renin–angiotensin–aldosterone system (RAAS) inhibitor drugs and COVID-19 [20]. Gao et al. study [21], which included 2877 patients [29.5% (850/2877) had a history of hypertension], showed that patients with hypertension had a two-fold increase in the relative risk of mortality as compared with patients without hypertension (*HR* = 2.12, 95% *CI*: 1.17–3.82, *P* = 0.013). The mortality rates were similar between the RAAS inhibitor and non-RAAS inhibitor cohorts (*HR* = 0.85, 95% *CI*: 0.28–2.58, *P* = 0.774). However, in a study-level meta-analysis of four studies, the result showed that patients with RAAS inhibitor use tend to have a lower risk of mortality (*RR* = 0.65, 95% *CI*: 0.45–0.94, *P* = 0.02) [21–24]. Besides, several studies [25–28] have shown that suffering from hypertension is related to COVID-19 morbidity, mortality and so on. However, we have not found any research on the direct relationship between hypertension and recurrent positive. As for monocyte count (percentage), we also found an interaction (*P* = 0.002, 0.036, respectively). The higher the monocyte count (percentage), the lower the risk of recurrent positive. Unfortunately, we also did not find clinical studies related to this. Gibellini et al.[29] research indicated that compared with the healthy control group, COVID-19’s patients showed impaired of functional and bioenergetics on monocytes. The impairment was that monocytes had broad defects in metabolic pathways, not only failing to increase glycolysis but also exhibiting reduced oxygen consumption rate, together with important mitochondrial dysfunction. From the phenotypic point of view, the upregulation of inhibitory checkpoints, including PD-1 and PD-L1.

There are some limitations in our study. First, this study is a case-control study, including unavoidable potential confounders; therefore, we used strict statistical adjustment to minimize residual confounding. Second, as the study population contains only Chinese participants, it may be not generalizable to other ethnic groups. Third, all controls (non-recurrent positive) were followed up for only two months, and it was not clear whether they will return to positive after that, but the current study showed that most recurrent occur within 1 month, rarely more than two months[5, 19].

## Conclusions

In conclusion, SARS-CoV-2 RNA positive duration was associated with a higher risk of recurrent positive and especially SPD more than 28 day had a two-fold increase in the relative risk of re-positive as compared with SPD less than 14 day. What’s more, the risk may be higher among those with hypertension and lower monocyte count or percentage.

## Supplementary Information

**Additional file1:** **Table S1.** Clinical classification of COVID-19 according to Chinese COVID-19 prevention and treatment guidelines (8th edition). **Table S2.** No. of missing values and non-missing values. **Table S3.** Comparison of missing variables before and after multiple imputation.

## Data Availability

The data used or analysed during the current study are available from the corresponding author on reasonable request.

## Abbreviations

SPD: SARS-CoV-2 RNA positive duration
*OR*: Odds ratio
IQR: Interquartile range
SD: Standard deviation
CHD: Coronary heart disease
CPD: Chronic pulmonary disease
CKD: Chronic kidney disease
CLD: Chronic liver disease
WBC: White blood cell count
NC: Neutrophil count
NP: Neutrophil percentage
LC: Lymphocyte count
LP: Lymphocyte percentage
MC: Monocyte count
MP: Monocyte percentage
PLT: Platelet count
*CI*: Confidence interval
Ref.: Reference
GAM: Generalized additive model
