# Supplementary material for: The relationship between SARS-COV-2 RNA positive duration and the risk of recurrent positive
Source: Infect Dis Poverty. 2021 Mar 31;10:45. doi: 10.1186/s40249-021-00831-6 (PMC8010778; doi:10.1186/s40249-021-00831-6)
Supplement: Supplementary file 1 — Additional file1: Table S1. Clinical classification of COVID-19 according to Chinese COVID-19 prevention and treatment guidelines (8th edition). Table S2. No. of missing values and non-missing values. Table S3. Comparison of missing variables before and after multiple imputation. [file 40249_2021_831_MOESM1_ESM.docx]

**Table S1. Clinical classification of COVID-19 according to Chinese COVID-19 prevention and treatment guidelines (8^th^ edition)**

| **Mild type** |
| --- |
| The clinical symptoms were mild and no pneumonia was found in imaging. |
| **Moderate type** |
| The patient has symptoms such as fever and respiratory tract, and pneumonia can be seen on imaging. |
| **Severe type** |
| Adults who meet any of the following criteria can be diagnosed as severe COVID-19: |
| (1) Shortness of breath and respiratory rate > 30 beats / min |
| (2) In a resting state, oxygen saturation ≤ 93%. |
| (3) PaO_2_ / Fi0_2_ ≤ 300mmHg |
| (4) Lung imaging showed that the lung lesions progressed significantly (>50%) within 24-48 hours |
| **Critical type** |
| Adults who meet any of the following criteria can be diagnosed as critical COVID-19: |
| (1) Respiratory failure occurs and requires mechanical ventilation |
| (2) Shock occurs |
| (3) Patients with other organ failure need ICU monitoring and treatment. |

**Table S2 No. of missing values and non-missing values**

| Variables | No. of non-missing values | No. of missing values |
| --- | --- | --- |
| Sex | 411 | 0 |
| Age | 408 | 3 |
| BMI | 342 | 69 |
| Hypertension | 411 | 0 |
| Diabetes | 411 | 0 |
| Coronary heart disease | 411 | 0 |
| Chronic pulmonary disease | 411 | 0 |
| Chronic kidney disease | 411 | 0 |
| No. of chronic diseases | 411 | 0 |
| Malignant tumor | 411 | 0 |
| Chronic diseases | 411 | 0 |
| Clinical type | 410 | 1 |
| No. of antiviral drugs | 411 | 0 |
| Glucocorticoid | 411 | 0 |
| White blood cell count | 395 | 16 |
| Neutrophil count | 395 | 16 |
| Neutrophil percentage | 395 | 16 |
| Lymphocyte count | 395 | 16 |
| Lymphocyte percentage | 395 | 16 |
| Monocyte count | 395 | 16 |
| Monocyte percentage | 395 | 16 |
| Hemoglobin | 371 | 40 |
| Platelet count | 340 | 71 |

**Table S3 Comparison of missing variables before and after multiple imputation.**

|  | Data imputation before | Data imputation after | P value |
| --- | --- | --- | --- |
| Age | 48.7 (17.1)  49.0 (37.0-62.0) | 51.1 (48.5)  49.0 (37.0-62.0) | 0.335 |
| BMI | 22.9 (3.3)  22.9 (21.1-24.8) | 23.0 (3.9)  22.7 (21.0-24.8) | 0.921 |
| White blood cell count | 5.8 (2.3)  5.3 (4.5-6.8) | 5.9 (2.4)  5.4 (4.5-6.9) | 0.597 |
| Neutrophil count | 3.3 (1.6)  3.0 (2.3-4.2) | 3.3 (1.6)  3.0 (2.3-4.1) | 0.949 |
| Neutrophil percentage | 57.1 (15.7)  59.3 (51.6-67.2) | 56.5 (15.9)  58.7 (50.2-66.8) | 0.551 |
| Lymphocyte count | 1.7 (1.0)  1.5 (1.2-2.0) | 1.7 (1.0)  1.5 (1.2-2.0) | 0.913 |
| Lymphocyte percentage | 31.3 (13.6)  28.9 (22.1-36.2) | 30.9 (13.6)  28.5 (21.5-35.8) | 0.701 |
| Monocyte count | 0.5 (0.3)  0.4 (0.3-0.6) | 0.5 (0.3)  0.4 (0.3-0.6) | 0.971 |
| Monocyte percentage | 9.5 (5.0)  8.3 (6.6-10.8) | 9.5 (4.9)  8.3 (6.6-10.8) | 0.844 |
| Hemoglobin | 129.0 (17.0)  129.0 (119.0-139.0) | 129.2 (19.4)  129.0 (117.9-139.0) | 0.856 |
| Platelet count | 211.3 (70.5)  198.0 (166.8-242.0) | 212.5 (71.8)  200.0 (166.0-244.0) | 0.813 |
| Clinical type |  |  | 0.999 |
| Mild | 28 (6.8%) | 29 (7.1%) |  |
| Moderate | 320 (78.0%) | 320 (77.9%) |  |
| Severe | 49 (12.0%) | 49 (11.9%) |  |
| Critical | 13 (3.2%) | 13 (3.2%) |  |

Continuous variables are expressed as Mean (SD) and Median (Q1-Q3), while categorical variables are expressed as frequency and percentage.
